# Clinical Implications of Cardiac Symptoms and Electrocardiographic Abnormalities for Advanced Liver Fibrosis in Patients with Nonalcoholic Fatty Liver Disease

**DOI:** 10.3390/medicina59020375

**Published:** 2023-02-15

**Authors:** Min-Kyu Kang, Min-Cheol Kim

**Affiliations:** Department of Internal Medicine, College of Medicine, Yeungnam University, Daegu 712-749, Republic of Korea

**Keywords:** nonalcoholic fatty liver disease (NALFD), cardiac symptoms, electrocardiography (ECG), NAFLD fibrosis score (NFS), fibrosis-4 index (Fib-4)

## Abstract

*Background and Objectives*: Advanced liver fibrosis in patients with nonalcoholic fatty liver disease (NAFLD) can be a major predictor of cardiovascular disease (CVD) events and cardiac complications. However, the clinical significance of cardiac symptoms and abnormal electrocardiography (ECG) findings in patients with NAFLD associated with advanced liver fibrosis is unclear. Therefore, our study was aimed to evaluate the clinical implications based on the association between cardiac symptoms with ECG abnormalities for advanced liver fibrosis in patients with NAFLD. *Materials and Methods*: Of 31,795 participants who underwent health checkups, 6293 were diagnosed with NAFLD using ultrasound and inclusion criteria in a retrospective cross-sectional study. Advanced liver fibrosis was assessed based on a low NAFLD fibrosis score (NFS) and fibrosis-4 index (Fib-4) cut-off values (COVs). Cardiac data were assessed using a cardiac symptom questionnaire and 12-lead electrocardiography (ECG). *Results*: Among 6293 NAFLD patients with NAFLD, 304 (4.8%) experienced cardiac symptoms. NFS and Fib-4 indicated higher rates of advanced fibrosis in the cardiac-symptomatic group than in the non-symptomatic group (NFS: 7.3 vs. 4.1%; Fib-4: 7.8 vs. 3.7%; both *p* < 0.001). Cardiac symptoms were independently associated with advanced liver fibrosis using a step-wise-adjusted model and NFS and Fib-4 (final adjusted odds ratio (aOR), 1.40; 95% CI, 1.06–1.85; *p* = 0.018 for NFS; aOR, 1.67; 95%, 1.30–2.15; *p* < 0.001 for Fib-4). Cardiac symptoms with abnormal ECG findings independently predicted advanced liver fibrosis (aOR, 2.43; 95% CI, 1.72–3.39; *p* < 0.001 for NFS; aOR, 3.02; 95% CI, 2.19–4.15; *p* < 0.001 for Fib-4). *Conclusions*: Patients who have had cardiac symptoms and some ECG abnormalities may have a higher association with advanced liver fibrosis.

## 1. Introduction

The prevalence of nonalcoholic fatty liver disease (NAFLD) is approximately 20–30% in Korea, as well as in Western countries, and is rapidly increasing worldwide, with a predicted prevalence of 55.7% [1,2,3]. NAFLD comprises a wide spectrum of chronic liver diseases, including simple steatosis, steatohepatitis, advanced fibrosis, and cirrhotic changes [4]. Fibrosis is known to be strongly associated with all-cause and cardiovascular mortality in patients with NAFLD [5,6]. Noninvasive fibrosis scoring systems, such as the NAFLD fibrosis score (NFS) and fibrosis-4 index (Fib-4), which have negative predictive values > 90%, are the most suitable first-line tools for clinically ruling out advanced fibrosis in primary healthcare or tertiary referral centers [7,8]. Recent guidelines demand referral to a tertiary or liver center in possible cases of intermediate-to-high risk for advanced fibrosis based on low cut-off values (COVs) of Fib-4 and/or NFS in patients with NAFLD [7,8].

In patients with NAFLD, cardiovascular disease (CVD) is the predominant cause of mortality, and advanced fibrosis is a major predictor of CVD events and cardiac complications [9,10,11,12]. A recent meta-analysis showed that NAFLD is strongly associated with an approximately 1.5-fold increased risk of severe cardiac complications, including myocardial infarction, ischemic stroke, atrial fibrillation, and heart failure [13]. Therefore, patients with NAFLD who have new-onset symptoms related to CVD should be referred to a cardiologist for further cardiac evaluation, including electrocardiography (ECG), cardiac stress tests, and echocardiography [14]. Nevertheless, the clinical significance of cardiac symptoms and abnormal ECG findings in patients with advanced liver fibrosis and NAFLD remains poorly understood.

Herein, we investigated the association of advanced fibrosis and cardiac symptoms with abnormal ECGs using a simple questionnaire and a standard 12-lead ECG in patients with NAFLD.

## 2. Materials and Methods

### 2.1. Enrolled Participants

**Figure 1:**
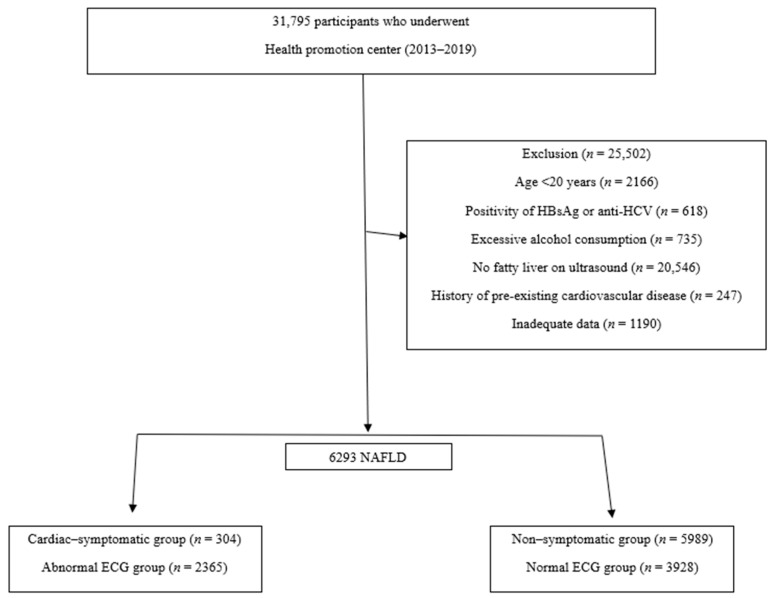
Flowchart of the enrolled patients.

Between September 2013 and June 2019, 31,795 participants who underwent health screening evaluations at Yeungnam University Hospital Health Promotion Center were eligible for inclusion in this retrospective cross-sectional study. A total of 25,502 participants were excluded based on the following criteria: (1) age < 20 years (*n* = 2166); (2) positivity for HBsAg or anti-HCV (*n* = 618); (3) history of heavy drinking (males, >140 g/week; females, >70 g/week; *n* = 735); (4) no advanced evidence of fatty liver on abdominal ultrasound (*n* = 20,546); (5) history of pre-existing CVD (*n* = 247); and (6) inadequate or missing data (*n* = 1190). Therefore, a total of 6293 patients with NAFLD were enrolled in this study. The study protocol was approved by the Institutional Review Board of Yeungnam University Hospital (IRB No. 2020-03-027). The requirement for informed consent was waived because of the retrospective study design (Figure 1).

### 2.2. Acquisition and Definition of Clinical Variables

Trained nurses obtained anthropometric data, including body mass index (BMI), seated blood pressure (BP), and waist circumference (WC). Fasting blood samples were collected, and abdominal ultrasound (US) was performed after a 12 h overnight fast. Serologic data, including aspartate aminotransferase (AST), alanine aminotransferase (ALT), γ-glutamyl transferase (GGT), albumin, high-sensitivity C-reactive protein (hsCRP), fasting blood sugar (FBS), total cholesterol (TC), triglyceride (TG), and high-density lipoprotein cholesterol (HDL-C) levels, and platelet counts, were evaluated in this study.

The four comorbidities in this study were defined as follows: obesity was defined as a BMI ≥ 25 kg/m^2^, in accordance with the Asian-Pacific criteria [15]. Type 2 diabetes mellitus (T2DM) was defined as FBS ≥ 126 mg/dL and the use of antidiabetic medications in accordance with the criteria of the American Diabetes Association [16]. Hypertension was defined as systolic BP ≥ 140 mmHg, diastolic BP ≥ 90 mmHg, or the use of antihypertensive medications [17]. Metabolic syndrome was defined as the presence of at least two of the following: WC ≥ 90 cm in men and ≥80 cm in women, BP ≥ 130/85 mmHg or being prescribed antihypertensive medication, TG ≥ 150 mg/dL, HDL-C level ≤ 40 mg/dL in men and ≤ 50 mg/dL in women, or an elevated fasting plasma glucose (FPG) level ≥ 100 mg/dL [18].

### 2.3. Definition of Nonalcoholic Fatty Liver Disease and Advanced Fibrosis

Hepatic steatosis was defined based on abdominal US findings (EPIQ 5 and EPIQ 7 (Philips, Amsterdam, The Netherlands)), in accordance with the Asia-Pacific guidelines [19]. NAFLD was defined as fatty liver that satisfied the exclusion criteria of the Asia-Pacific Working Party on NAFLD guidelines [20].

Given that the low COVs of NFS (−1.455) and Fib-4 (1.30) were strong negative predictors of advanced liver fibrosis in patients with NAFLD, advanced liver fibrosis was defined as having a NAFLD fibrosis score and fibrosis-4 index above the low NFS and Fib-4 COVs, respectively [7,8]. The two noninvasive fibrosis scoring systems used were as follows: NFS, −1.675 + 0.037 × age (years) + 0.094 × BMI (kg/m^2^) + 1.13 × impaired FBS/T2DM (yes = 1, no = 0) + 0.99 × AST/ALT ratio—0.013 × platelet count (×10^9^/L)—0.66 × albumin (g/dL); Fib-4, age (years) × AST (U/L)/(platelet count [10^9^/L] × ALT [U/L]) [21,22].

### 2.4. Assessment of ECG Abnormalities and Cardiac Symptoms

Based on a 12-lead ECG using the GE MAC 5000 system (GE Health Care, Chicago, IL, USA), normal ECG findings were defined as a regular sinus rhythm of 60–100 beats per min without any abnormal P-wave, PR interval, QRS complex, ST segment, T-wave, or QT interval findings. These were confirmed by a cardiologist with 10 years of experience who was blinded to the patient’s clinical manifestations [23]. If the normal ECG finding was unsatisfactory, it was defined as an abnormal ECG. Major cardiac symptoms were classified into the following six categories: chest discomfort, palpitations, constricting chest pain, shortness of breath, near fainting (presyncope), and dizziness within 1 year. In cases with multiple cardiac symptoms, the most uncomfortable symptom was used as the standard (Supplementary Table S1).

### 2.5. Statistical Analysis

Continuous variables are shown as mean ± standard deviation, and categorical variables are presented as numbers (%). Differences in the variables according to the presence or absence of cardiac symptoms in patients with NAFLD were evaluated using the Student’s *t*-test. The association between ECG abnormalities, cardiac symptoms, and advanced fibrosis in patients with NAFLD was assessed using a logistic regression analysis. Except for the variables used to calculate NFS and Fib-4, the classic risk factors for NAFLD were analyzed in sequentially adjusted models. All analyses were performed using R statistical software (version 4.1.0; R Foundation for Statistical Computing, Vienna, Austria), and statistical significance was set at *p* < 0.05.

## 3. Results

### 3.1. Baseline Demographic and Clinical Characteristics of the Participants

The baseline demographic and clinical characteristics of the participants, based on the presence or absence of cardiac symptoms, are shown in Table 1. Among the 6293 patients with NAFLD, 304 (4.8%) had cardiac symptoms. Participants with cardiac symptoms (symptom group) were older (57.4 vs. 51.8 years), had a higher BMI (25.6 vs. 25.2 kg/m^2^) and WC (85.5 vs. 84.0 cm), and had a higher prevalence of abnormal ECG findings (54.9 vs. 36.7%) and atrial fibrillation (6.6 vs. 0.7%) than did those without cardiac symptoms (non-symptom group). Abnormal ECG findings such as ST depression, which is considered an indicator of ischemic heart disease, were not observed in this study. The most common cardiac symptoms were chest discomfort (47.4%), palpitations (14.5%), chest pain (12.8%), shortness of breath (11.2%), presyncope (7.2%), and dizziness (6.9%). Platelet counts and HDL levels were higher in the symptomatic group than in the non-symptomatic group. Additionally, the percentages of advanced fibrosis, as defined by the NFS and Fib-4, were higher in the symptomatic group than in the non-symptomatic group.

### 3.2. Percentage of Cardiac Manifestations According to the Presence or Absence of Advanced Liver Fibrosis, as Defined by Two Fibrosis Scoring Systems

**Figure 2:**
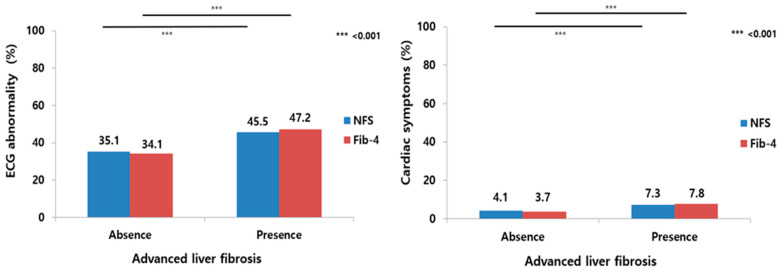
The percentage differences of ECG abnormalities (**left**) and cardiac symptoms (**right**), according to the presence or absence of advanced liver fibrosis using two fibrosis scoring systems in patients with nonalcoholic fatty liver disease. ECG, electrocardiogram; NFS, nonalcoholic fatty liver disease fibrosis score; Fib-4, fibrosis-4 index.

The percentage of ECG abnormalities was 35.1% in the absence of advanced fibrosis; 45.5% in the presence of advanced fibrosis, as defined by the NFS; 34.1% in the absence of advanced fibrosis; and 47.2% in the presence of advanced fibrosis, as defined by Fib-4 (*p* < 0.001). Moreover, the percentage of cardiac symptoms was 4.1% in the absence of advanced fibrosis; 7.3% in the presence of advanced fibrosis, as defined by NFS; 3.7% in the absence of advanced fibrosis; and 7.8% in the presence of advanced fibrosis, as defined by Fib-4 (*p* < 0.001; Figure 2).

### 3.3. Effects of ECG Abnormalities and Cardiac Symptoms on Advanced Liver Fibrosis in Patients with NAFLD

We investigated the association of ECG abnormalities and cardiac symptoms with advanced fibrosis using a multivariable-adjusted model. Table 2 shows the adjusted odds ratios (ORs) of ECG abnormalities and the presence of cardiac symptoms for advanced fibrosis, as defined by the NFS and Fib-4. To avoid multicollinearity, age, BMI, the presence of diabetes mellitus, and levels of AST, ALT, platelets, and albumin, which were used to calculate the NFS, were excluded from use as variables in the multivariable model. Additionally, age and AST, ALT, and platelet levels, which were used to calculate Fib-4, were excluded from use as variables in the multivariable model.

The association between ECG abnormalities and advanced fibrosis identified using the NFS remained significant after adjusting for sex, hypertension, and obesity (model 1: OR, 1.35; 95% confidence interval [CI], 1.18–1.54). The association after the step-wise addition of FPG and hsCRP (model 2: OR, 1.40; 95% CI, 1.23–1.60), TC, TG, HDL-C, and GGT (model 3: OR, 1.38; 95% CI, 1.21–1.58) was not attenuated. The association between ECG abnormalities and advanced fibrosis identified using Fib-4 remained significant after adjusting for sex, T2DM, hypertension, and obesity (model 1: OR, 1.64; 95% CI, 1.45–1.85). The associations after the step-wise addition of TC, TG, and HDL-C (model 2: OR, 1.63; 95% CI, 1.44–1.84) and FPG, albumin, GGT, and hsCRP (model 3: OR, 1.63; 95% CI, 1.44–1.85) were not attenuated. In addition, considering that ECG abnormalities were associated with aging and diabetes mellitus, ECG abnormalities were associated with advanced liver fibrosis based on NFS and Fib-4 after the adjusted model using age and diabetes mellitus (Supplementary Table S2).

Moreover, the association between the presence of cardiac symptoms and advanced fibrosis identified using the NFS persisted after adjusting for sex, hypertension, and obesity (model 1: OR, 1.44; 95% CI, 1.10–1.88). After further additions of FPG and hsCRP (model 2: OR, 1.44; 95% CI, 1.09–1.89) and TC, TG, HDL-C, and GGT (model 3: OR, 1.40; 95% CI, 1.06–1.85), the association remained significant. The association between the presence of cardiac symptoms and advanced fibrosis identified using Fib-4 persisted after adjusting for sex, T2DM, hypertension, and obesity (model 1: OR, 1.72; 95% CI, 1.34–2.21; *p* < 0.001). The association remained significant after further additions of TC, TG, and HDL-C (model 2: OR, 1.67; 95% CI, 1.30–2.15; *p* < 0.001) and FPG, albumin, GGT, and hsCRP (model 3: OR, 1.67; 95% CI, 1.30–2.15; *p* < 0.001).

### 3.4. Variables Associated with Advanced Liver Fibrosis for Cardiac Symptoms with ECG Abnormalities in Patients with NAFLD

Considering that abnormal ECGs and cardiac symptoms have complementary characteristics, we investigated the effects of cardiac symptoms with ECG abnormalities on advanced fibrosis in patients with NAFLD. Among 6293 patients, 3791 had normal ECGs with no cardiac symptoms, while 167 had cardiac symptoms with abnormal ECG findings.

Table 3 shows the variables associated with advanced fibrosis, as defined by NFS and Fib-4, in patients with NAFLD. In the multivariable analysis, hypertension (OR, 1.87; 95% CI, 1.53–2.28; *p* < 0.001), obesity (OR, 2.20; 95% CI, 1.86–2.61; *p* < 0.001), FBS (OR, 7.11; 95% CI, 5.17–9.88; *p* < 0.001), hsCRP (OR, 1.01; 95% CI, 1.01–1.03; *p* = 0.028), and the presence of cardiac symptoms with abnormal ECGs findings (OR, 2.36; 95% CI, 1.67–3.31; *p* < 0.001) were associated with advanced liver fibrosis, as defined by the NFS in patients with NAFLD. Additionally, T2DM (OR, 1.54; 95% CI, 1.21–1.95; *p* < 0.001), hypertension (OR, 1.57; 95% CI, 1.29–1.90; *p* < 0.001), albumin levels (OR, 0.69; 95% CI, 0.53–0.92; *p* = 0.011), and the presence of cardiac symptoms with abnormal ECGs (OR, 3.02; 95% CI, 2.19–4.15; *p* < 0.001) were associated with advanced liver fibrosis, as defined by the Fib-4 in patients with NAFLD (Table 3).

### 3.5. Step-Wise Adjustment to Identify the Association of Cardiac Symptoms with Abnormal ECGs and Advanced Fibrosis in Patients with NAFLD

Cardiac symptoms with abnormal ECGs in advanced liver fibrosis, as defined by the NFS, remained significant after adjusting for sex, hypertension, and obesity (model 1: OR, 2.45; 95% CI, 1.76–3.38). The associations remained significant after the step-wise additions of FPG and hsCRP (model 2: OR, 2.49; 95% CI, 1.78–3.47) and TC, TG, HDL-C, and GGT (model 3: OR, 2.43; 95% CI, 1.72–3.39). Additionally, cardiac symptoms with abnormal ECGs in advanced liver fibrosis, as defined by the Fib-4, persisted after adjusting for sex, T2DM, hypertension, and obesity (model 1: OR, 3.15; 95% CI, 2.30–4.33). The association remained significant after the step-wise addition of TC, TG, and HDL-C (model 2: OR, 3.09; 95% CI, 2.25–4.25) and FBS, albumin, GGT, and hsCRP (model 3: OR, 3.02; 95% CI, 2.19–4.15) (Table 4).

## 4. Discussion

To our knowledge, this is the first study to evaluate the association between cardiac symptoms and abnormal ECG findings in patients with NAFLD and advanced liver fibrosis. Regardless of which of the two noninvasive fibrosis scoring systems is applied, the components and combinations of cardiac symptoms and abnormal ECG findings may be associated with advanced fibrosis, independent of step-wise adjustments for traditional risk factors.

NAFLD is the hepatic manifestation of multiple organ cross-talk due to shared mechanisms, including insulin resistance and chronic inflammation factors such as tumor necrosis factor-alpha and interleukin-6, which contribute to increased CVD events and risk of cardiovascular mortality [12,24,25]. NAFLD is well known to be associated with an increased risk of subclinical atherosclerosis; fatal and non-fatal CVD events; and other cardiac complications, including arrhythmia, cardiac dysfunction, and valvular heart disease [12,26,27,28,29]. Recently, significant or advanced liver fibrosis in patients with NAFLD was found to be strongly associated with CVD events [30,31,32]. Park et al. demonstrated that significant fibrosis, as defined by a magnetic resonance elastography stiffness ≥ 2.97 kPa, was independently associated with the presence of coronary artery calcification using an age- and sex-adjusted model (aOR = 3.21–3.53; *p* < 0.05) [32]. Additionally, Han et al. demonstrated that significant fibrosis, as defined by the NFS and Fib-4, was associated with an increased risk of atherosclerotic CVD in patients with NAFLD (aOR = 2.38; *p* < 0.001) [30]. In a meta-analysis, Fib-4 and NFS, rather than the AST-to-platelet ratio index, might be a significant noninvasive fibrosis scoring system for estimating higher CVD events in patients with NAFLD [33]. In another study, high liver fibrosis scores, as defined by low FIB-4 and NFS COVs, were associated with an approximately two-fold increased adjusted risk of CVD events (cardiovascular death, nonfatal myocardial infarction, and ischemic stroke) in patients who underwent coronary interventions [31]. However, the clinical significance of cardiac symptoms and/or abnormal ECG findings in terms of advanced liver fibrosis in patients with NAFLD remains unclear. In the current study, the presence of cardiac symptoms with abnormal ECG findings was associated with an approximately 2.5-fold increased adjusted risk of advanced fibrosis, as defined by low FIB-4 and NFS COVs, in patients with NAFLD.

Based on recent NAFLD guidelines and expert opinions, the Fib-4 and NFS serve as first-line tests that can be used to assess the need for referral to liver specialists in primary care [7,8]. The possibility of having an intermediate-to-high risk of advanced liver fibrosis should be assessed using low FIB-4 and NFS COVs; subsequently, referral to a center may be necessary for further fibrosis assessment [7]. Despite the simplicity and repeatability of Fib-4 and NFS, physicians should not blindly use noninvasive fibrosis scoring systems as their only decision-making tool [8,34]. The formal components of Fib-4 and NFS contain only indirect markers of anthropometric variables (age, BMI, and diabetes), liver damage (AST and ALT), and evidence of portal hypertension (platelet counts) fibrosis assessments [8]. Additionally, the algorithms of these tools do not include any cardiac evaluation, such as cardiovascular testing or questionnaires. Therefore, the identification of new cardiovascular risk factors associated with fibrosis in primary care remains necessary. Given the association between cardiac symptoms, abnormal ECG findings, and advanced liver fibrosis in our results, a simple questionnaire on cardiac symptoms and the use of 12-lead ECGs may be used as additional tools for predicting advanced liver fibrosis in patients with NAFLD in primary care.

The findings of our study should be interpreted carefully due to the following limitations. First, due to the single-center retrospective nature of the study, the causal relationship between cardiac symptoms and advanced liver fibrosis in patients with NAFLD was not fully elucidated. Second, owing to the possibility of selection and recall biases, the participants included in the current study may not be representative of the population. A possible source of selection bias in this study was that the participants were examinees who were concerned about their health and could afford to pay for medical expenses. While there is a possibility of recall bias for the presence of cardiac symptoms within one year, cardiac symptoms are well known to be closely associated with serious complications, making them more memorable than other symptoms. Moreover, participants concerned about their health would be expected to remember such symptoms. Third, due to the qualitative evaluation of the cardiac symptom questionnaire used in our study, it is limited in its ability to reveal clear associations between advanced fibrosis and cardiac comorbidities in patients with NAFLD. Gastroesophageal reflux disease and other pulmonary diseases that could be mistaken for cardiac symptoms were not excluded and may have been evaluated in our study. Fourth, owing to the ambiguity of the definition of abnormal ECG, we could not evaluate the clinical impacts of specific types of abnormal ECGs. However, ECG findings do not always correlate with cardiac disease severity. Further research is needed to determine the clinical significance of well-classified specific ECG findings for advanced liver fibrosis in patients with NAFLD. Fifth, owing to the lack of homeostasis model assessments in our center, the association between IR and advanced liver fibrosis has not been elucidated. Finally, due to the lack of data on patients’ familial history of cardiac disease and smoking, we could not evaluate the clinical impact of these factors on advanced fibrosis in patients with NAFLD.

## 5. Conclusions

The presence of cardiac symptoms and abnormal ECG findings may be independently associated with advanced liver fibrosis in patients with NAFLD, regardless of classic metabolic factors. Our study showed the possibility of screening for simple cardiac data and advanced liver fibrosis in patients with NAFLD. In primary care, assessments using simple 12-lead ECGs and cardiac symptom questionnaires for patients who have NAFLD and possibly advanced liver fibrosis may be an alternative for liver center referral. However, prospective studies would be merited to explore this area further.

## Figures and Tables

**Table 1:** Baseline characteristics.

Variable	Cardiac Symptoms *n* = 304 (4.8%)	No Cardiac Symptoms *n* = 5989 (95.2%)	*p*-Value
Age (yr)	57.4 ± 9.8	51.8 ± 9.3	<0.001
Male, *n* (%)	177 (58.2)	3486 (58.2)	0.975
BMI, kg/m^2^	25.6 ± 2.7	25.2 ± 2.6	0.025
WC (cm)	85.5 ± 7.5	84.0 ± 7.3	<0.001
Comorbidities			
Obesity, *n* (%)	163 (53.6)	2886 (48.2)	0.074
Diabetes mellitus, *n* (%)	36 (11.8)	560 (9.4)	0.178
Hypertension, *n* (%)	58 (19.1)	984 (16.4)	0.257
Metabolic syndrome, *n* (%)	64 (21.1)	1109 (18.5)	0.302
ECG findings			
ECG abnormality, *n* (%)	167 (54.9)	2198 (36.7)	<0.001
Atrial fibrillation, *n* (%)	20 (6.6)	39 (0.7)	<0.001
Major cardiac symptoms			
Chest discomfort, *n* (%)	144 (47.4)		
Palpitation, *n* (%)	44 (14.5)		
Chest pain, *n* (%)	39 (12.8)		
Shortness of breath, *n* (%)	34 (11.2)		
Near fainting (presyncope), *n* (%)	22 (7.2)		
Dizziness, *n* (%)	21 (6.9)		
Liver profile			
AST, IU/L	27.8 ± 12.8	26.9 ± 12.3	0.223
ALT, IU/L	30.5 ± 18.6	31.2 ± 20.5	0.474
PLT, K/uL	238.5 ± 53.8	249.3 ± 58.2	0.002
GGT, IU/L	33.7 ± 24.8	36.0 ± 37.7	0.117
Albumin, g/dL	4.7 ± 0.3	4.7 ± 0.6	0.069
Metabolic profiles			
FPG, mg/dL	102.7 ± 20.3	101.6 ± 24.7	0.392
TC, mg/dL	205.4 ± 44.9	208.5 ± 39.0	0.246
TG, mg/dL	148.4 ± 99.7	150.7 ± 91.1	0.698
HDL, mg/dL	55.4 ± 14.0	53.7 ± 13.5	0.031
LDL, mg/dL	120.3 ± 42.5	124.7 ± 36.7	0.082
hsCRP, mg/dL	0.15 ± 0.25	0.13 ± 0.30	0.073
Fibrosis scoring system			
NAFLD fibrosis score	−1.8 ± 1.1	−2.3 ± 1.1	<0.001
Fibrosis-4 index	1.3 ± 0.6	1.1 ± 0.5	<0.001
Advanced fibrosis			
NAFLD fibrosis score, *n* (%)	78 (25.7)	1127 (18.8)	0.004
Fibrosis-4 index, *n* (%)	98 (32.2)	1274 (21.3)	<0.001

Values are shown as mean ± standard deviation or number (%). BMI, body mass index; WC, waist circumference; ECG, electrocardiogram; AST, aspartate aminotransferase; ALT, alanine aminotransferase; PLT, platelet count; GGT, gamma-glutamyl transferase; FPG, fasting plasma glucose; TC, total cholesterol; TG, triglyceride; HDL, high-density lipoprotein; LDL, low-density lipoprotein; hsCRP, high-sensitivity C-reactive protein; NAFLD, nonalcoholic fatty liver disease.

**Table 2:** Adjusted odds ratios of electrocardiogram abnormalities and the presence of cardiac symptoms for advanced liver fibrosis defined and identified using the NFS and Fib-4.

	ECG Abnormality		Cardiac Symptoms	
	OR (95% CI)	*p*-Value	OR (95% CI)	*p*-Value
OR for advanced fibrosis by NFS				
Unadjusted	1.37 (1.21–1.56)	<0.001	1.49 (1.14–1.94)	0.003
Sex adjusted	1.35 (1.19–1.53)	<0.001	1.49 (1.14–1.94)	0.003
Model 1	1.35 (1.18–1.54)	<0.001	1.44 (1.10–1.88)	0.008
Model 2	1.40 (1.23–1.60)	<0.001	1.44 (1.09–1.89)	0.009
Model 3	1.38 (1.21–1.58)	<0.001	1.40 (1.06–1.85)	0.018
OR for advanced fibrosis by Fib-4				
Unadjusted	1.63 (1.45–1.84)	<0.001	1.76 (1.37–2.26)	<0.001
Sex adjusted	1.63 (1.45–1.84)	<0.001	1.76 (1.37–2.26)	<0.001
Model 1	1.64 (1.45–1.85)	<0.001	1.72 (1.34–2.21)	<0.001
Model 2	1.63 (1.44–1.84)	<0.001	1.67 (1.30–2.15)	<0.001
Model 3	1.63 (1.44–1.85)	<0.001	1.67 (1.30–2.15)	<0.001

NAFLD, nonalcoholic fatty liver disease; ECG, electrocardiogram; OR, odds ratio; CI, confidence interval; NFS, NAFLD fibrosis score; Fib-4, fibrosis-4 index. Adjusted models using the NFS. Model 1: Sex, presence of hypertension, and obesity. Model 2: Further adjusted for fasting plasma glucose and high-sensitivity C-reactive protein. Model 3: Further adjusted for total cholesterol, triglyceride, high-density lipoprotein cholesterol, and gamma-glutamyl transferase. Adjusted models using the Fib-4. Model 1: Sex, presence of diabetes, hypertension, and obesity. Model 2: Further adjusted for total cholesterol, triglyceride, and high-density lipoprotein cholesterol. Model 3: Further adjusted for fasting plasma glucose, albumin, gamma-glutamyl transferase, and high-sensitivity C-reactive protein. All variables in models 2 and 3 used continuous values.

**Table 3:** Multivariable analysis of variables associated with advanced fibrosis in patients with nonalcoholic fatty liver disease.

Variables	Advanced Fibrosis Using NFS *				Advanced Fibrosis Using Fib-4 ^†^			
	Univariate *p*-Value	Multivariable			Univariate *p*-Value	Multivariable		
		OR	95% CI	*p*-Value		OR	95% CI	*p*-Value
Age, years *^,†^	<0.001				<0.001			
Male	0.398				0.103			
T2DM *					<0.001	1.54	1.21–1.95	<0.001
Hypertension	<0.001	1.87	1.53–2.28	<0.001	<0.001	1.57	1.29–1.90	<0.001
Obesity	<0.001	2.20	1.86–2.61	<0.001	0.012			
FBS, mg/dL	<0.001	7.11	5.17–9.88	<0.001	0.002			
GGT, U/L	0.321				0.221			
Albumin, g/dL *					<0.001	0.69	0.53–0.92	0.011
hsCRP, mg/L	0.001	1.01	1.00–1.03	0.028	0.300			
TC, mg/dL	<0.001				<0.001			
TG, mg/dL	0.281				0.072			
HDL-C, mg/dL	0.002				0.333			
Symptoms with abnormal ECG	<0.001	2.36	1.67–3.31	<0.001	<0.001	3.02	2.19–4.15	<0.001

NFS, nonalcoholic fatty liver disease fibrosis score; Fib-4, fibrosis-4 index; OR, odds ratio; CI, confidence interval; T2DM, type 2 diabetes mellitus; FBS, fasting blood sugar; GGT, gamma-glutamyl transferase; hsCRP, high-sensitivity C-reactive protein; TC, total cholesterol; TG, triglyceride; HDL-C, high-density lipoprotein cholesterol; ECG, electrocardiogram. * To avoid multicollinearity, age, BMI level, presence of diabetes mellitus, and aspartate aminotransferase, alanine aminotransferase, platelet, and albumin levels, which were used to calculate the NFS, were excluded from use as variables in the multivariable model. Additionally, **^†^** age and aspartate aminotransferase, alanine aminotransferase, and platelet levels, which were used to calculate Fib-4, were excluded from use as variables in the multivariable model.

**Table 4:** Adjusted odds ratios of the presence of cardiac symptoms with abnormal electrocardiograms for advanced liver fibrosis defined using the NAFLD fibrosis score and fibrosis-4 index.

	Cardiac Symptoms with Abnormal ECG	
	OR (95% CI)	*p*-Value
OR for advanced fibrosis by NFS		
Unadjusted	2.59 (1.88–3.56)	<0.001
Gender adjusted	2.59 (1.88–3.56)	<0.001
Model 1	2.45 (1.76–3.38)	<0.001
Model 2	2.49 (1.78–3.47)	<0.001
Model 3	2.43 (1.72–3.39)	<0.001
OR for advanced fibrosis by Fib-4		
Unadjusted	3.20 (2.33–4.37)	<0.001
Gender adjusted	3.22 (2.35–4.41)	<0.001
Model 1	3.15 (2.30–4.33)	<0.001
Model 2	3.09 (2.25–4.25)	<0.001
Model 3	3.02 (2.19–4.15)	<0.001

NAFLD, nonalcoholic fatty liver disease; ECG, electrocardiogram; OR, odds ratio; CI, confidence interval; COV, cut-off value; NFS, NAFLD fibrosis score. Adjusted models using the NFS. Model 1: Sex, presence of hypertension, and obesity. Model 2: Further adjusted for fasting plasma glucose and high-sensitivity C-reactive protein. Model 3: Further adjusted for total cholesterol, triglyceride, high-density lipoprotein cholesterol, and gamma-glutamyl transferase. Adjusted models using the Fib-4. Model 1: Sex, presence of diabetes, hypertension, and obesity. Model 2: Further adjusted for total cholesterol, triglyceride, and high-density lipoprotein cholesterol. Model 3: Further adjusted for fasting plasma glucose, albumin, gamma-glutamyl transferase, and high-sensitivity C-reactive protein. All variables in models 2 and 3 used continuous values.

## Data Availability

The data used to support the findings of this study are available from the corresponding author upon request.

## Supplementary Materials

The following supporting information can be downloaded at: https://www.mdpi.com/article/10.3390/medicina59020375/s1, Table S1: Cardiac symptoms questionnaire; Table S2: Age and DM-adjusted odds ratios of ECG abnormalities for advanced liver fibrosis defined and identified using the NFS and Fib-4.
