# Supplementary material for: Clinical Implications of Cardiac Symptoms and Electrocardiographic Abnormalities for Advanced Liver Fibrosis in Patients with Nonalcoholic Fatty Liver Disease
Source: Medicina (Kaunas). 2023 Feb 15;59(2):375. doi: 10.3390/medicina59020375 (PMC9959224; doi:10.3390/medicina59020375)
Supplement: Supplementary file 1 [file medicina-59-00375-s001.zip › medicina-2227227-supplementary.pdf]

Supplementary Table S1. Cardiac symptoms questionnaire

Please indicate √ in the appropriate column.	Yes	No
1. Have you ever experienced chest discomfort within the past year?		
2. Have you experienced palpitations within the past year?		
3. Have you ever experienced squeezing-nature chest pain within the past year?		
4. Have you ever experienced shortness of breath during exercise within the past year?		
5. Have you experienced near-fainting within the past year?		
6. Have you experienced dizziness within the past year?		

Supplementary Table S2. Age and DM-adjusted odds ratios of ECG abnormalities for advanced liver fibrosis defined and identified using the NFS and Fib-4

	ECG abnormalities	
	OR (95% CI)	P-value
OR for advanced fibrosis by NFS		
Unadjusted	1.37 (1.21-1.56)	<0.001
Age and DM adjusted	1.23 (1.08-1.41)	0.002
OR for advanced fibrosis by Fib-4		
Unadjusted	1.63 (1.45–1.84)	<0.001
Age and DM adjusted	1.31 (1.15–1.49)	<0.001

ECG, electrocardiogram; OR, odds ratio, CI, confidence interval; DM, NFS, NAFLD fibrosis score; Fib-4, fibrosis-4 index; DM, diabetes mellitus.
